# Tissue optimization strategies for high‐quality ex vivo diffusion imaging

**DOI:** 10.1002/nbm.4866

**Published:** 2022-12-04

**Authors:** Rachel L. C. Barrett, Diana Cash, Camilla Simmons, Eugene Kim, Tobias C. Wood, Richard Stones, Anthony C. Vernon, Marco Catani, Flavio Dell'Acqua

**Affiliations:** ^1^ NatBrainLab, Institute of Psychiatry, Psychology and Neuroscience, King's College London UK; ^2^ Department of Neuroimaging, Institute of Psychiatry, Psychology and Neuroscience, King's College London UK; ^3^ Sackler Institute for Translational Neurodevelopment, Department of Forensic and Neurodevelopmental Science, Institute of Psychiatry, Psychology and Neuroscience, King's College London UK; ^4^ Department of Basic and Clinical Neuroscience, Institute of Psychiatry, Psychology, and Neuroscience, King's College London UK

**Keywords:** diffusion MRI, ex vivo, fixed tissue preparation, high resolution, SNR efficiency, tractography

## Abstract

Ex vivo diffusion imaging can be used to study healthy and pathological tissue microstructure in the rodent brain with high resolution, providing a link between in vivo MRI and ex vivo microscopy techniques. Major challenges for the successful acquisition of ex vivo diffusion imaging data however are changes in the relaxivity and diffusivity of brain tissue following perfusion fixation.

In this study we address this question by examining the combined effects of tissue preparation factors that influence signal‐to‐noise ratio (SNR) and consequently image quality, including fixative concentration, contrast agent concentration and tissue rehydration time. We present an optimization strategy combining these factors to manipulate the $T_{1}$ and $T_{2}$ of fixed tissue and maximize SNR efficiency.

We apply this strategy in the rat brain, for a diffusion‐weighted spin echo protocol with TE = 27 ms on a 9.4 T scanner with a 39 mm volume coil and 660 mT/m 114 mm gradient insert. We used a reduced fixative concentration of 2% paraformaldehyde (PFA), rehydration time more than 20 days, 15 mM Gd‐DTPA in perfusate and TR 250 ms. This resulted in a doubling of SNR and an increase in SNR per unit time of 135% in cortical grey matter and 88% in white matter compared with 4% PFA and no contrast agent.

This improved SNR efficiency enabled the acquisition of excellent‐quality high‐resolution (78 μm isotropic voxel size) diffusion data with *b* = 4000 s/mm ${}^{2}$, 30 diffusion directions and a field of view of 40×13×18 mm^3^ in less than 4 days. It was also possible to achieve comparable data quality for a standard resolution (150 μm) diffusion dataset in $2\frac{1}{4}$ h.

In conclusion, the tissue optimization strategy presented here may be used to improve SNR, increase spatial resolution and/or allow faster acquisitions in preclinical ex vivo diffusion MRI experiments.

## Graphical abstract

We present a tissue preparation optimization strategy for ex vivo MRI in the rat brain. Tissue parameters, fixative concentration, gadolinium and rehydration time are used to alter $T_{1}$ and $T_{2}$ and are optimized with scanning parameters to improve SNR efficiency. Tissue optimization resulted in double the SNR compared with a standard preparation. We used this method to acquire high‐resolution HARDI diffusion MRI data and to explore detailed anatomical features with tractography.

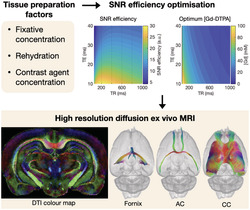

**Abbreviations**
AC: anterior commissure
CC: corpus callosum
DTI: diffusion tensor imaging
EPI: echo planar imaging
FA: fractional anisotropy
Gd‐DTPA: gadopentetic acid
HARDI: high‐angular‐resolution diffusion imaging
MD: mean diffusivity
PFA: paraformaldehyde
PBS: phosphate buffered saline
RARE: rapid acquisition with relaxation enhancement
SNR: signal‐to‐noise ratio
TE: echo time
TR: repetition time

## 1 INTRODUCTION

High‐resolution ex vivo diffusion MRI offers unique opportunities to study tissue microstructure and three‐dimensional organization of fibres in the healthy rodent brain and in models relevant for human brain disorders. Ex vivo experiments allow for longer scan times than in vivo, enabling higher spatial resolution to be achieved. Due to changes in brain tissue properties after death and fixation, however, ex vivo MRI in general and diffusion imaging in particular have challenges in achieving a sufficient signal‐to‐noise ratio (SNR).^1^, ^2^, ^3^, ^4^, ^5^, ^6^, ^7^

Among the changes associated with tissue fixation are reduced diffusivity^2^, ^6^, ^8^, ^9^ and decreases in $T_{1}$ and $T_{2}$ relaxation time.^1^, ^2^, ^10^, ^11^, ^12^, ^13^, ^14^, ^15^ Reduced diffusivity requires more diffusion weighting to achieve adequate contrast,^2^, ^4^ particularly for advanced diffusion models.^16^ Higher diffusion weighting in general requires a longer echo time (TE), hence signal loss from $T_{2}$ relaxation becomes a limiting factor.

Another limiting factor for high‐resolution ex vivo diffusion imaging is scan time. Large acquisition matrices are required for high spatial resolution, which increases the acquisition time of a single volume. Multiple volumes are needed for sampling different diffusion directions; e.g., for high‐angular‐resolution diffusion imaging (HARDI), 30–60 directions are recommended.^16^, ^17^, ^18^, ^19^ As there is a trade‐off between SNR, voxel size and scan time, the time required to achieve adequate signal (e.g. SNR >10 in non‐diffusion‐weighted images) for ex vivo high‐spatial‐ and angular‐resolution diffusion imaging quickly becomes prohibitive unless a strategy is used to improve SNR with respect to scan time, or SNR efficiency.

This paper focuses on tissue preparation factors that can be optimized to boost SNR efficiency. Optimized tissue preparation can be used with other approaches for improving tissue SNR efficiency such as hardware and pulse sequence optimization, to further improve results. Equally, it can be applied to improve SNR efficiency and enhance the possibilities of high‐resolution imaging in the absence of optimal hardware or advanced pulse sequences, as we demonstrate in this paper.

In this study we varied three tissue preparation factors to manipulate the $T_{1}$ and $T_{2}$ characteristics of fixed rat brain tissue and maximize SNR efficiency: fixative concentration, rehydration or soaking in phosphate buffered saline (PBS) and the concentration of gadolinium introduced into the tissue with the ‘active staining’ technique.^20^, ^21^ Lower fixative concentrations^15^, ^22^, ^23^ and rehydration^2^, ^13^, ^14^, ^15^, ^24^, ^25^ are associated with prolonging $T_{2}$ relaxation time, and are therefore used to improve SNR. The addition of gadolinium to the tissue primarily reduces $T_{1}$ relaxation time, allowing the repetition time (TR) to be shortened without losing signal due to incomplete longitudinal relaxation.^2^, ^20^, ^26^, ^27^ As $T_{2}$ relaxation time is also reduced in gadolinium‐stained tissue, however, the concentration must be optimized to maximize SNR efficiency. We have made available an interactive tool for SNR efficiency optimisation at https://github.com/rachellcb/SNR-Efficiency-Calculator.

A quantitative optimization of gadolinium concentration in terms of SNR efficiency has previously been shown in the macaque brain in Reference.^2^ To our knowledge, the present study is the first to include fixative as well as contrast agent concentration as variables, to present SNR efficiency optimization data for tissue fixed at 2% paraformaldehyde (PFA) and to quantify changes in relaxation constants over time immersed in PBS.

We start by measuring the effect of the tissue preparation factors on relaxation and diffusion properties and modelling how they can be combined to maximize SNR efficiency. We then demonstrate the resulting improvement in SNR by comparing data from optimized versus standard tissue preparations. Finally, using the optimized tissue, we present examples of high‐resolution and more conventional diffusion data acquisitions.

## 2 MATERIALS AND METHODS

### 2.1 Animal preparation

Animal experiments were conducted with approval from the local King's College London ethics committee in accordance with the UK Home Office Animals (Scientific Procedures) Act 1986. Adult male rats (Sprague Dawley, n=30) were euthanized with pentobarbital (60 mg/kg i.p.) and transcardially perfused for approximately two minutes at 100 mL/min pump speed with a minimum of 200 mL ice‐cold heparinized 0.9% saline (50 IU/mL) followed by a minimum of 200 mL of either 2% or 4% PFA (‘Parafix’, Pioneer Research Chemicals, Colchester, UK) buffered at 7.4 pH containing gadopentetic acid (Gd‐DTPA) in a concentration range of 0–50 mM. After perfusion, the heads were removed and immersed in the fixation mixture for four days. We chose a post‐fixation period of four days to ensure adequate fixation when experimenting with the reduced fixative concentration; however, it may be sufficient to post‐fix for shorter periods (e.g., 24 h or less), as done elsewhere in rats fixed with the standard fixative concentration.^28^, ^29^

The samples were then rehydrated in 50 mL PBS with 0.05% sodium azide preservative and either 0 or 1 mM Gd‐DTPA (Magnevist, Bayer, Reading, UK). For comparison two additional samples were prepared similarly using gadobutrol (Gadovist, Bayer, Reading, UK), as at the time of writing gadobutrol is set to replace Gd‐DTPA in some clinical settings. Details of the fixative concentration, contrast agent concentration and rehydration time for all samples are summarized in Table 1. Following the literature,^20^, ^21^, ^28^, ^30^, ^31^ we used higher concentrations of contrast agent in the perfusion stage followed by a lower concentration in the rehydration stage, as the blood–brain barrier inhibits the contrast agent from entering the tissue before fixation, whereas the tissue becomes more permeable to contrast agent after fixation.^32^

**TABLE 1:** Rat preparation parameters for rehydration and gadolinium experiments, optimized high‐resolution diffusion protocol and contrast agent comparison

Experiment	n	[Fixative ]	[CA] in perfusate	[CA] in rehydration solution	Rehydration interval (days)
Rehydration experiment	4	4% ( n=2), 2% ( n=2)			0, 2, 4, 6, 14, 24, 35 (scanned sequentially)
Gadolinium experiment	20	4%, 2% ( n=2 for each [CA])	0, 15, 25, 35, 50 mM Gd‐DTPA	1 mM Gd‐DTPA	35–38*
Reference (no gadolinium)	2	4% ( n=1), 2% ( n=1)			35
High‐ and mid‐resolution diffusion	1	2%	15 mM Gd‐DTPA	1 mM Gd‐DTPA	≤ 35
Contrast agent comparison	4	4%, 2% ( n=2 for each [CA])	5, 15 mM gadobutrol	1 mM gadobutrol	35

*Note*: n, number of rats used for each experiment; CA, contrast agent. *Rats perfused with 35 mM Gd‐DTPA were rehydrated for 55–56 days.

All samples were refrigerated at 4 ° C during rehydration, as a precaution against tissue degradation given the lower fixative concentration used in some cases. Samples were moved to room temperature 4 h before scanning. Brains remained in situ for the rehydration and gadolinium experiments, where spatial resolution was not critical, to reduce the risk of tissue damage during skull removal and handling. Excess tissue was trimmed from around the skull. For the high‐resolution acquisition, where a smaller field of view was critical to minimize scan time, the brain was carefully removed from the skull directly prior to scanning. For scanning, samples were sealed in plastic tubes padded with gauze to prevent movement and immersed in proton‐free fluorinated liquid (Galden; Solvay, Watford, UK) to reduce susceptibility artefacts.

### 2.2 MRI hardware

MRI was performed at the BRAIN Centre (brain‐imaging.org), King's College London, on a 9.4 T Bruker (Ettlingen, Germany) BioSpec scanner, with a 39 mm volume coil (Rapid Biomedical, Rimpar, Germany). The rehydration, gadolinium and contrast agent comparison experiments, and the mid‐resolution diffusion acquisition were carried out with a 660 mT/m 114 mm gradient set. The high‐resolution diffusion acquisition was carried out using a 1000 mT/m 60 mm gradient insert.

### 2.3 Relaxometry

$T_{1}$ maps were acquired using a 2D rapid acquisition with relaxation enhancement (RARE) sequence with variable TR = 200, 400, 800, 1500, 3000 and 5500 ms, TE = 7 ms, RARE factor = 2, acquisition matrix = 128×128×7 and voxel size = 0.22×0.22×1.00 mm^3^. $T_{2}$ maps were acquired using a multi‐slice multi‐echo sequence with TR = 2000 ms, TE = 8, 16, 24, 32, 40, 48, 56, 64, 72, 80, 88, 96, 104, 112, 120, 128, 136, 144, 152, 160, 168 and 176 ms, and the same geometric parameters as used for $T_{1}$ mapping.

**FIGURE 1:**
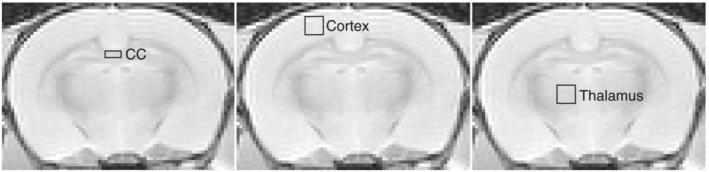
Regions of interest in the CC, cortex and thalamus used in the rehydration and gadolinium experiments

$T_{1}$ and $T_{2}$ relaxation parameters were estimated using non‐linear least squares curve fitting in MATLAB (version 9.6.0, MathWorks, Natick, MA, USA). The average $T_{1}$ and $T_{2}$ within regions of interest in the corpus callosum (CC), cortex and thalamus were calculated to compare samples (Figure 1). For the rehydration experiment, $T_{1}$ and $T_{2}$ values were plotted against rehydration time. For the gadolinium experiment, $T_{1}$ and $T_{2}$ were modelled with respect to the concentration of contrast agent according to the relationship^33^

(1) $$\frac{1}{T_{i}}=\frac{1}{T_{i0}}+r_{i}[\text{CA}],\;i=1,2,$$

where $T_{i}$ is the observed relaxation time, $T_{i0}$ is the baseline relaxation time, which in this case represents the tissue soaked in PBS with 1 mM contrast agent, $r_{i}$ is the relaxivity of the contrast agent in the tissue and [CA] refers to the concentration of contrast agent added to the perfusate during fixation.

### 2.4 DTI measurements for optimization

For the rehydration and gadolinium experiments, diffusion tensor imaging (DTI) measurements were carried out using a 2D diffusion‐weighted echo planar imaging (EPI) sequence with a target *b*‐value of 1500 s/mm ${}^{2}$, 30 diffusion‐weighted and 5 non‐diffusion‐weighted images, TR = 1000 ms, TE = 21 ms, four segments, acquisition matrix = 128×128×7 and voxel size = 0.23×0.23×1.00 mm^3^. The data were denoised using Marchenko–Pastur principal component analysis noise estimation for diffusion MRI^34^ and Gibbs‐ringing correction as described in Reference.^35^ The data were then corrected for eddy current distortions and diffusion tensor parameters were estimated using ExploreDTI 4.8.6 (www.exploredti.com). Fractional anisotropy (FA) and mean diffusivity (MD) were recorded for regions of interest in the CC, cortex and thalamus and plotted against rehydration time or [Gd‐DTPA].

### 2.5 SNR efficiency simulation

SNR efficiency was calculated in terms of $T_{1}$, $T_{2}$, TR and TE, where $T_{1}$ and $T_{2}$ were modelled in terms of [Gd‐DTPA] as described above using Equation (1). SNR efficiency is defined as $\frac{1}{\sqrt{T_{R}}}$SNR and given by the formula^2^

(2) $${\text{SNR}}_{\text{eff}}\propto \frac{1}{\sqrt{TR}}e^{\frac{-TE}{T_{2}}}\left(1-e^{\frac{-TR}{T_{1}}}\left(2e^{\frac{-TE}{2T_{2}}}-1\right)\right),$$

based on the signal equation for a standard spin echo sequence. This allowed us to predict SNR efficiency for any given combination of [Gd‐DTPA], TR and TE. This SNR efficiency optimisation tool is available at https://github.com/rachellcb/SNR-Efficiency-Calculator. This simulation was used to determine the amount of contrast agent (15 mM Gd‐DTPA) used in the sample optimized for the high‐resolution diffusion acquisition described below.

### 2.6 SNR comparison

To compare SNR between standard and optimized tissue preparations, one rat brain fixed with 4% fixative and no gadolinium, rehydrated in PBS, was compared with another fixed with 2% fixative and 15 mM Gd‐DTPA, rehydrated in PBS with 1 mM Gd‐DTPA. A 3D diffusion‐weighted spin echo acquisition was used with one diffusion‐weighted volume (*b* = 2500 s/mm ${}^{2}$) and one non‐diffusion‐weighted volume, with TR = 250 ms, TE = 26.82 ms, flip angle = 90°, acquisition matrix = 104×93×8 and isotropic voxel size = 0.25 mm, and scan time of 6.2 min. Data from both samples were acquired in the same session.

Mean signal ( η) values were recorded for regions of interest in the CC and cortex, and the standard deviation of the noise ( $\sigma_{\text{noise}}$) was measured from a region outside the sample. SNR was then estimated using the formula from Reference^36^:

$$\text{SNR}=\sqrt{2-\frac{\pi }{2}}\frac{\eta }{\sigma_{\text{noise}}}\approx 0.655\frac{\eta }{\sigma_{\text{noise}}}.$$

All SNR measurements in this paper were calculated from data without or prior to denoising.

### 2.7 High‐ and mid‐resolution diffusion protocols

The high‐resolution example was acquired using a 3D diffusion‐weighted spin echo sequence. We chose an isotropic voxel size of 78 μm, a b‐value of 4000 s/mm ${}^{2}$ and 30 diffusion‐weighted directions (plus three non‐diffusion‐weighted volumes) to ensure adequate diffusion contrast for tractography^4^, ^19^ and a maximum scan time of 96 h to illustrate an example of a protocol that can practically be acquired over 4 days or a long weekend (the actual scan time was 91.35 h or 2.77 h per volume). Within these constraints, we chose the minimum TE, 26.78 ms, and optimal TR, 250 ms, in line with SNR efficiency considerations. We used δ = 3 ms, Δ = 11 ms, maximum gradient amplitude = 577 mT/m, flip angle = 90° and acquisition matrix = 512×162×232.

The mid‐resolution example diffusion protocol was acquired using 3D diffusion‐weighted EPI with TR = 280 ms, TE = 26.16 ms, *b* = 2000 s/mm ${}^{2},\;\delta$ = 4 ms, Δ = 12.5 ms, gradient amplitude 405 mT/m, flip angle = 90°, eight segments, four averages, 30 diffusion‐ and 5 non‐diffusion‐weighted images, isotropic voxel size = 150 μm, acquisition matrix = 108×80×108 and scan time = 9.13 h (16.6 min per volume). The EPI data were processed as described in Section 2.4.

The high‐resolution spin echo dataset was denoised as described above. As spin echo data are robust to eddy current and geometric distortions, no further corrections were required. Diffusion modelling and tractography processing were done using StarTrack (https://www.natbrainlab.co.uk). Whole brain tractography data were generated using both DTI and spherical deconvolution models. For DTI tractography, a stopping threshold of FA = 0.1, angle threshold 45° and step size 78 μm were used. For spherical deconvolution, the fibre orientation distribution function was estimated using the damped Richardson–Lucy algorithm described in Reference^37^ with parameters α = 0.6, n = 0.001, r = 8 and 500 iterations. The tractography stopping threshold for this model was hindrance modulated orientational anisotropy (HMOA) = 0.002, angle 45° and step size 78 μm, as above. Tracts were dissected in TrackVis 6.0.1 (http://www.trackvis.org) using manually drawn regions of interest with inclusion and exclusion conditions. The fornix and anterior commissure (AC) were reconstructed using DTI tractography and the CC using spherical deconvolution tractography because of its ability to resolve multiple fibre directions per voxel, for example where callosal and cortico‐spinal fibres cross. The quality of the diffusion and tractography data was assessed by visual inspection for the ability to resolve anatomical features. Anatomical validity of tracts was established with reference to axonal tracing atlases.^38^, ^39^

### 2.8 Histology

Histology and immunohistochemistry were performed to test whether reducing the fixative concentration or adding Gd‐DTPA to the fixation and rehydration stage had an impact on the quality of the fixed tissue. Three samples were compared, one fixed with 4% PFA, no contrast agent, i.e. the standard protocol, one fixed with 2% PFA, no contrast agent, to test the effect of reduced fixative concentration and one fixed with 2% PFA and 15 mM Gd‐DTPA to test the effect of active staining with a gadolinium contrast agent. Once scanning had been completed, the brains were extracted from the skulls, cryoprotected in 30% sucrose and sectioned at 35 μm thickness. Tissue sections were stained with Cresyl violet for Nissl bodies in neurons, Luxol fast blue for myelin or IBA‐1 antibody using immunohistochemistry testing for microglia. Slides from all three stains were scanned with an Olympus[S8] (Tokyo, Japan) VS120 slide scanner at ×40 magnification. The results were compared by inspection of expected cell and tissue morphologies, according to experience of previously immuno‐stained tissues using the same protocols.

## 3 RESULTS

### 3.1 Rehydration experiment

**FIGURE 2:**
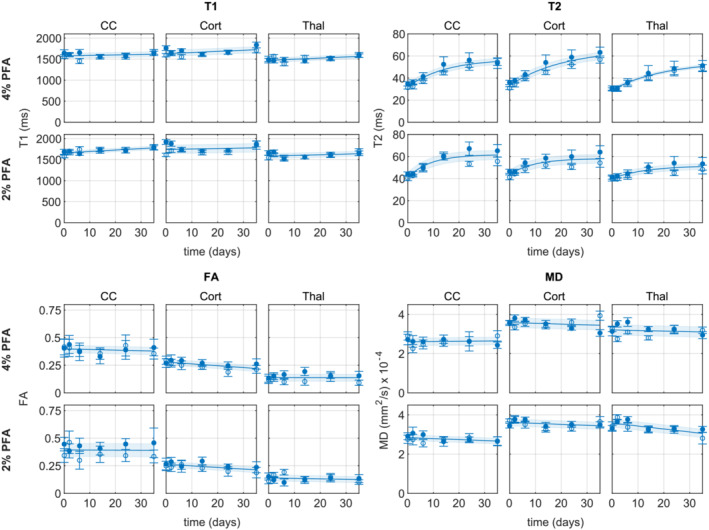
Plots showing how $T_{1}$, $T_{2}$, FA and MD vary with number of days soaking in PBS for rat heads fixed with 4% and 2% fixative in regions of interest in the CC white matter, cortical grey matter (Cort) and thalamus (Thal). Data points and error bars show mean ± standard deviation within the region of interest for a single rat. Open/closed circles correspond to individual rats. Lines of best fit ± standard error of the regression are shown

In this experiment we investigated the rehydration of fixed rat brains for 35 days in a single volume of PBS, with measurements in the CC, cortical grey matter and thalamus. Rehydration resulted in increased $T_{2}$ following an exponential recovery curve with relatively little or no effect on $T_{1}$ or diffusion metrics (Figure 2). $T_{1}$ increased by between 2 and 7% across the different regions of interest for both the 4% and 2% PFA models. $T_{2}$ increased by 72–80% in the 4% PFA model, and 27–50% in the 2% model. The initial $T_{2}$ of the 4% model (29–32 ms, across regions of interest) was lower than the 2% model (40–43 ms) and after 35 days the values converged (to 50–61 ms). T1, T2, FA and MD data for all experiments are available at https://doi.org/10.6084/m9.figshare.21629894.

$T_{2}$ stabilized faster for the 2% fixative model than the 4% model, taking 17 days in the CC, 14 days in the cortex and a projected 20 days in the thalamus to increase to within 95% of the model plateau value, compared with 32, 47 and 46 days projected in the 4% model for the CC, cortex and thalamus regions respectively.

The DTI metrics, FA and MD, were relatively stable over the 35 day period. In the CC white matter region, the FA model remained between 0.38 and 0.40 for both fixative concentrations, and the MD model between $2.6\times 10^{-4}$ and $2.8\times 10^{-4}$. Weak effects of decreasing FA were observed in the cortex for both fixative concentrations, and decreasing MD in the thalamus region for the 2% PFA model.

### 3.2 Gadolinium experiment

**FIGURE 3:**
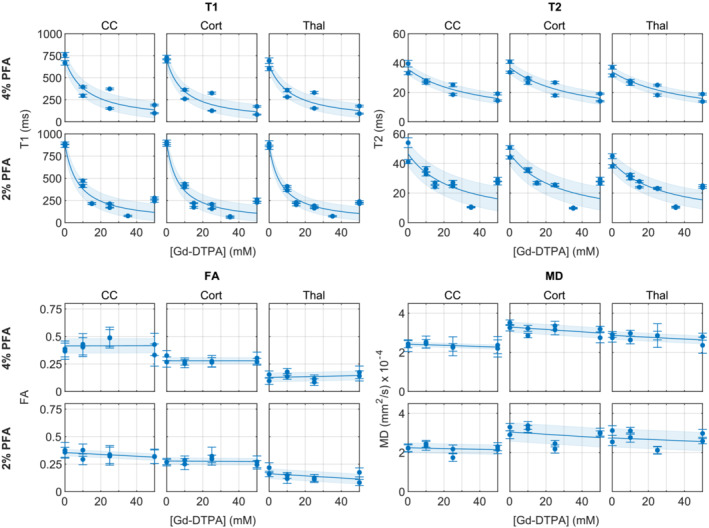
Plots showing how $T_{1}$, $T_{2}$, FA and MD vary with the concentration of Magnevist (Gd‐DTPA) used in perfusion for 4% and 2% fixative. As above, data points represent the mean ± standard deviation within the region of interest in the CC, cortical grey matter (Cort) and thalamus (Thal) for a single rat brain. $T_{1}$ and $T_{2}$ are modelled with Equation (1), FA and MD with lines of best fit. Shaded regions represent the standard error of the regression. All samples in this experiment, including those with no contrast agent in the perfusate, were rehydrated for at least 35 days in PBS with 1 mM Gd‐DTPA

This experiment shows the relationship of $T_{1}$, $T_{2}$ and diffusion properties to the concentration of Gd‐DTPA used in perfusion fixation (Figure 3). As expected, both $T_{1}$ and $T_{2}$ decrease with an inverse relationship to Gd‐DTPA concentration according to the model in Equation (1), with $T_{1}$ decreasing more rapidly than $T_{2}$. The estimated relaxation parameters for the model are given in Table 2. At lower concentrations, $T_{1}$ was greater in the 2% than the 4% PFA model, while the values converged at perfusion concentrations greater than about 10 mM. A similar pattern was seen in the $T_{2}$ results, with higher values for the 2% model up to about 50 mM. The results for the CC, cortical grey matter and thalamus region were very similar, with inter‐individual differences and the effect of contrast agent concentration being greater than intra‐individual tissue differences.

**TABLE 2:** Parameters used to model relaxation time with respect to contrast agent concentration, according to Equation (1)

Fixation	CA	Tissue	$T_{10}$	$r_{1[\text{CA}]}$	$\sigma_{1}$	$T_{20}$	$r_{2[\text{CA}]}$	$\sigma_{2}$
4% PFA	Gd‐DTPA	CC	707	0.120	93.4	35.8	0.703	3.50
		Cort	706	0.149	83.6	36.9	0.709	4.13
		Thal	643	0.127	78.6	34.0	0.672	3.31
2% PFA	Gd‐DTPA	CC	876	0.109	94.0	45.0	0.444	8.20
		Cort	896	0.167	90.6	46.8	0.801	7.75
		Thal	870	0.172	75.2	40.9	0.813	6.18
2% PFA	Gadobutrol	CC	354	0.267	67.1	30.4	0.438	2.90
		Cort	385	0.325	21.7	31.2	0.516	1.80
		Thal	353	0.301	39.5	28.7	0.603	2.31

*Note*: $T_{i0}$ is the baseline relaxation time for tissue fixed with no contrast agent and rehydrated in 1 mM contrast agent, units in ms; $r_{i}$ is the relaxivity constant for contrast agent added during fixation, units in 1/(mM s); and $\sigma_{i}$ is the standard error of the regression for the model fitting, i=1,2, units in ms. For Gd‐DTPA, n=10 for each fixative concentration. For the gadobutrol model, n=4. Abbreviations: CA, contrast agent; WM, white matter; GM, grey matter.

The initial values for $T_{1}$ and $T_{2}$ represent the baseline case with no contrast agent added to the perfusate, but still rehydrated in 1 mM Gd‐DTPA. For reference, we also measured $T_{1}$ and $T_{2}$ in rats with no contrast agent in either the perfusate or the rehydration solution. In the 4% fixative model with no contrast agent, $T_{1}$ values in the CC, cortex and thalamus respectively were 1705 ± 91 ms, 1760 ± 181 ms and 1607 ± 29 ms, and $T_{2}$ was 52 ± 2 ms, 51 ± 12 ms and 48 ± 4 ms ( n = 3). In the 2% fixative model, for the same regions respectively, $T_{1}$ was 1824 ± 63 ms, 1828 ± 81 ms and 1668 ± 27 ms and $T_{2}$ was 61 ± 5 ms, 57 ± 8 and 51 ± 3 ms ( n = 3).

The addition of contrast agent had relatively little effect on diffusion metrics. The model for FA in the 4% fixative case changed from 0.41 with no contrast agent in the fixative to 0.42 with 50 mM Gd‐DTPA, and in the 2% fixative case from 0.35 to 0.31. The MD model for 4% PFA changed from $2.42\times 10^{-4}$ to $2.27\times 10^{-4}$ mm ${}^{2}$/s, and in the 2% model from $2.25\times 10^{-4}$ to $2.14\times 10^{-4}$ mm ${}^{2}$/s.

Comparing contrast agents Gd‐DTPA and gadobutrol (Figure S1) revealed that $T_{1}$ was reduced in samples prepared with gadobutrol by a factor of 0.4–0.5. $T_{2}$ was shorter in gadobutrol samples by a factor of 0.7 initially, while the models for the two contrast agents converged at higher concentrations, particularly in the grey matter regions. The estimated parameters for the gadobutrol model are included in Table 2.

### 3.3 SNR efficiency optimization

**FIGURE 4:**
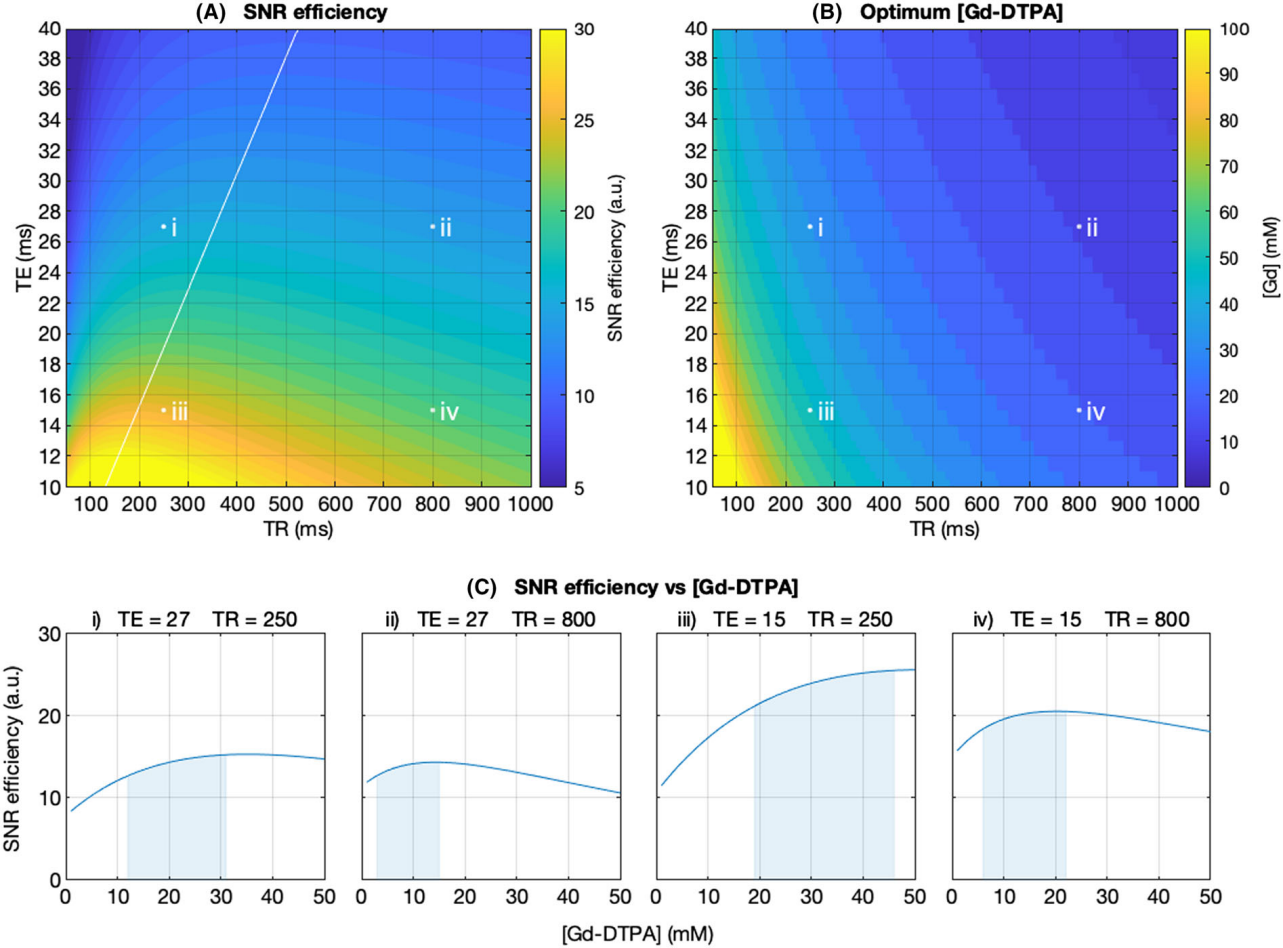
Plots showing how SNR efficiency varies with TE, TR and the concentration of Gd‐DTPA used in fixation. (A), SNR efficiency as a function of TE and TR, given the optimum [Gd‐DTPA] at each point. This plot can be used to determine the optimal TR for a given TE, following the white line. Values simulated using Equation 2, for SNR efficiency in spin echo sequences. (B), The [Gd‐DTPA] that gives maximum SNR efficiency for each combination of TE and TR. This plot is used to determine the optimal concentration of Gd‐DTPA for a given TE and TR. (C), SNR efficiency varying as a function of [Gd‐DTPA], for fixed values of TE and TR. Four examples are shown corresponding to points i–iv in A and B. Shaded regions indicate the [Gd‐DTPA] intervals resulting in 95% of the maximum SNR efficiency. The values shown in this figure were modelled using data from a region of interest in the CC in rats fixed with 2% PFA

The simulation of SNR efficiency based on the 2% PFA model can be used to find the optimum concentration of Gd‐DTPA, taking into consideration realistic constraints on TE and TR. Figure 4A shows the SNR efficiency for a range of TE and TR, given an optimal choice of Gd‐DTPA concentration. The optimal TR for a given TE is indicated in white. Figure 4B shows the concentrations of Gd‐DTPA that lead to maximum SNR efficiency, corresponding to Figure 4A. We observe that more contrast agent is required for shorter TE and TR and vice versa. To illustrate how SNR efficiency varies with contrast agent concentration for a given TE and TR, Figure 4C shows the SNR efficiency profiles for four selected examples: TE/TR = (i) 27/250 ms, (ii) 27/800 ms, (iii) 15/250 ms and (iv) 15/800 ms. As a guide for selecting the contrast agent concentration, the concentration range of Gd‐DTPA required to give within 5% of the maximum SNR efficiency is indicated in the shaded region, for the four example cases given in Figure 4. These are (i) 12–31 mM, (ii) 3–15 mM, (iii) 19–46 mM and (iv) 6–22 mM.

### 3.4 SNR comparison in standard versus optimized tissue

**FIGURE 5:**
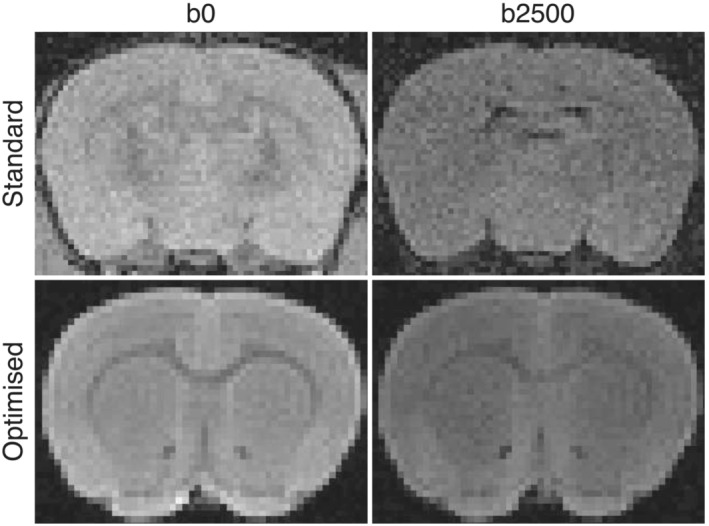
Improvement in SNR gained from optimizing tissue and scanning parameters. The standard preparation included [Gd‐DTPA] = 0 mM, [PFA] = 4% (top row). The optimized tissue preparation was fixed with [Gd‐DTPA] = 15 mM, [PFA] = 2% (bottom row). Images are shown for non‐diffusion‐weighted ( b 0) and diffusion‐weighted (*b*= 2500 s/mm ${}^{2}$) images, using a 3D spin echo pulse sequence. The optimized sample was removed from the skull in preparation for subsequent high‐resolution acquisition. Data from the two samples were acquired using the same coil and protocol

Here we compare SNR in a rat prepared using tissue optimized for the high‐resolution diffusion acquisition with a standard baseline case without tissue optimization (Figure 5). The standard sample, prepared without contrast agent and with the standard fixative concentration of 4% PFA, had SNR values of 12 and 10 in the cortex and CC white matter respectively in the non‐diffusion‐weighted image and 7 in both the CC and cortex in a diffusion‐weighted image with *b* = 2500 s/mm ${}^{2}$. The optimized sample, fixed with 2% PFA and 15 mM Gd‐DTPA, had SNR of 28 and 18 in cortical grey and CC white matter respectively in the non‐diffusion‐weighted and 18 and 16 respectively in the diffusion‐weighted volume. This represents an increase of SNR by a factor of 2.4 in cortical grey and 1.9 in CC white matter in the non‐diffusion‐weighted images. A visible reduction in noise can also be directly seen in the optimized images. In terms of SNR efficiency, the optimized protocol resulted in an SNR per unit time (in minutes) of 9 in cortical grey matter and 6 in CC white matter, compared with 4 and 3 in the standard sample, for the non‐diffusion‐weighted volume.

### 3.5 Example of high‐ and mid‐resolution diffusion imaging in optimized tissue

**FIGURE 6:**
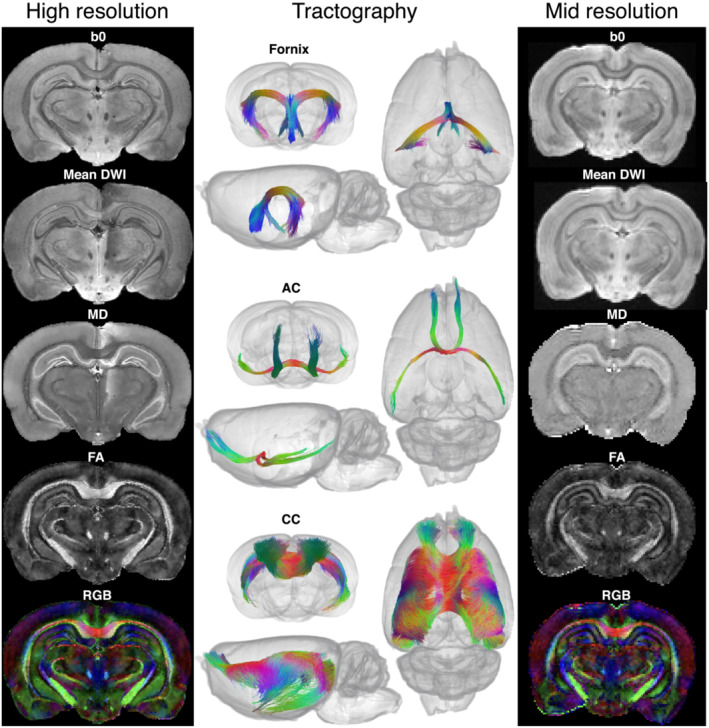
Diffusion data with optimized tissue preparation. Left, diffusion maps for the high‐resolution (78 μm) diffusion‐weighted spin echo acquisition, including a non‐diffusion‐weighted image (b0), the mean of all diffusion‐weighted images (Mean DWI), MD, FA and directional colour map with red–green–blue (RGB) encoding. Middle, tractography reconstructions of the fornix and fimbria, AC and CC using the high‐resolution data. Right, diffusion maps for the mid‐resolution (150 μm) diffusion‐weighted EPI acquisition. Images represent data from a single rat brain

**FIGURE 7:**
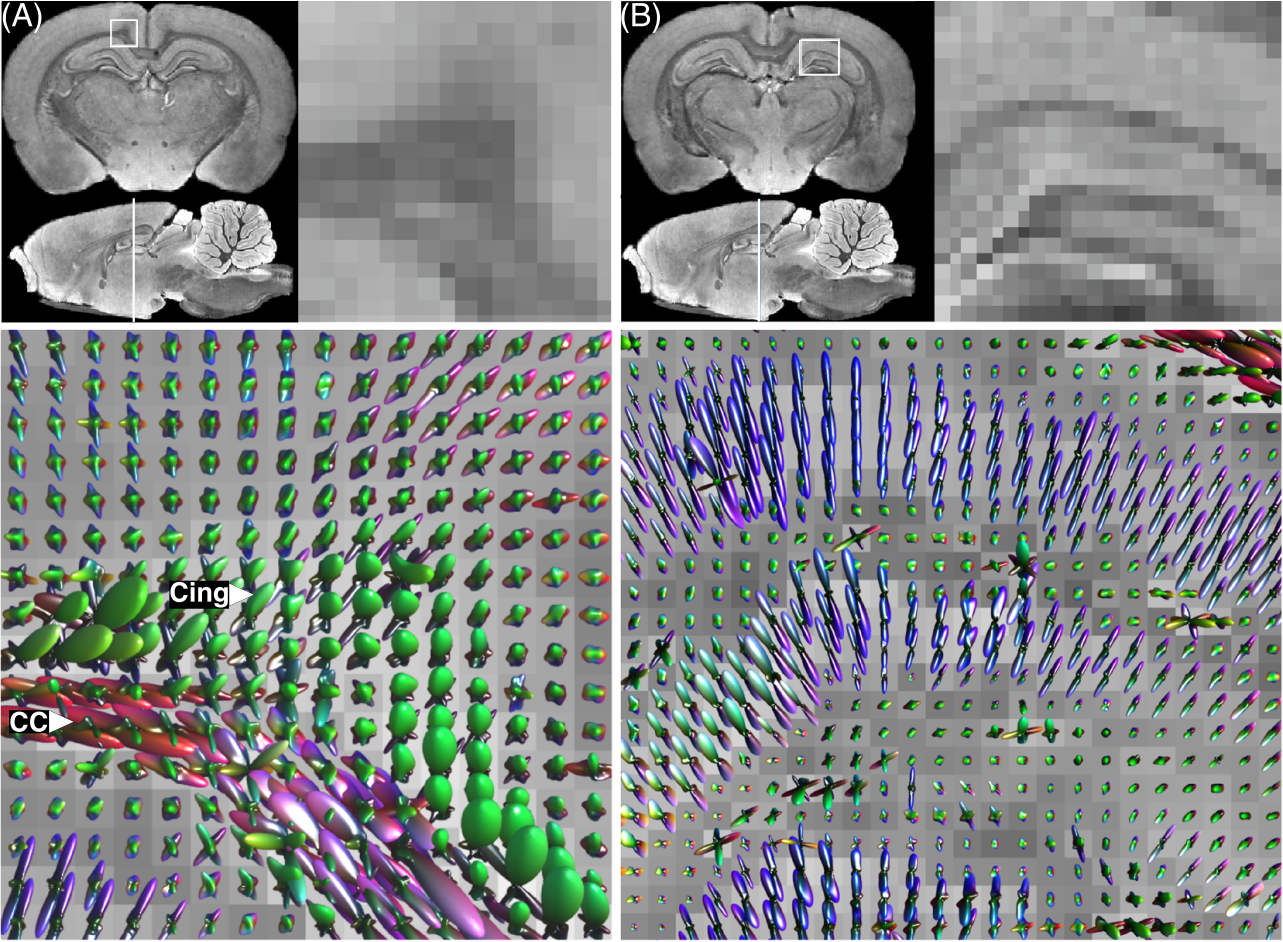
Fibre orientation distribution functions in high‐resolution diffusion data. Regions of interest shown are a region including crossing fibres of the CC and cingulum (Cing.) (A) and a region showing hippocampal layering (B). Top panels, region of interest location and enlarged view; bottom panels, fibre orientation distribution functions within the given region

These diffusion acquisitions serve as example applications of tissue optimization. In the high‐resolution acquisition, the FA was 0.55 in the middle of the CC and up to 0.7 elsewhere in the white matter. The DTI images and tractography reconstructions follow the known anatomy with a high level of detail, and fine anatomical structures are resolved (Figure 6). Layers of cell bodies in the hippocampus of about 50 μm thickness are visible in the DTI maps, and the diffusion characteristics of different hippocampal layers are modelled distinctly with fibre orientation distribution functions using spherical deconvolution (Figure 7). Small fibre bundles in the body and the lateral fimbria of the fornix, and posterior arms of the AC, were successfully reconstructed using tractography (Figure 6). The CC was reconstructed through crossing regions where it intersects with the cingulum (as illustrated in Figure 7).

The SNR before denoising of the non‐diffusion‐weighted images was 12 in a cortical grey matter region and 9 in the CC white matter. For comparison, we estimated that to achieve the same SNR with the standard sample preparation would require a TR of at least 750 ms, resulting in a tripling of the scan time to 11 days, 10 hours. This calculation assumes the same spatial resolution, field of view, TE and number of volumes, and is based on the parameters measured from samples fixed with 4% PFA, with no contrast agent, and 35 days rehydration in PBS ( $T_{1}$ = 1705 ms, $T_{2}$ = 52 ms).

For the mid‐resolution diffusion acquisition, the SNR in non‐diffusion‐weighted images was 23 in cortical grey and 17 in CC white matter. As SNR is proportional to the square root of the number of signal averages, we calculated that an adequate SNR for diffusion modelling, similar to that obtained in the high‐resolution dataset, can be achieved in 2^1/4^ h. Examples of diffusion‐ and non‐diffusion‐weighted images and DTI maps are included in Figure 6. Full datasets are available at https://doi.org/10.6084/m9.figshare.21629894.

### 3.6 Histology

The histology results showed no differences in quality between the standard protocol (4% fixative, no contrast agent), the standard protocol with reduced fixative concentration (2% fixative, no contrast agent) and our optimized protocol (Figure S2).

## 4 DISCUSSION

This study investigated how multiple tissue preparation factors (tissue rehydration time, fixative concentration and contrast agent concentration) can be combined to improve SNR efficiency for ex vivo diffusion MRI in the healthy adult male rat brain. As an application of this approach, we included an example of a high‐quality, high‐resolution diffusion protocol. The specific tissue preparation and scanning parameters used in this example may provide a valuable benchmark for future high‐resolution ex vivo diffusion experiments, and our optimization strategy in general may be used or adapted for a wide range of ex vivo MRI applications.

### 4.1 Rehydration experiment

As in previous studies, we found that rehydration increased the $T_{2}$ of fixed tissue and had comparatively little effect on $T_{1}$.^2^, ^15^, ^24^, ^25^ This increase in $T_{2}$ is thought to be due to the removal of unbound fixative from the tissue.^15^, ^24^ $T_{1}$ was expected to remain relatively stable, as the fixation reactions that cause a reduction of $T_{1}$ in fixed tissue are not reversed during rehydration.^13^, ^24^

In the 2% PFA model, the cortex was the first region to approach plateau in $T_{2}$, followed by the CC and the thalamus, although this effect of distance from the surface on rehydration rate was not observed as clearly in the 4% model. We would nonetheless recommend allowing a longer period for rehydration of deeper structures (e.g., 20 days or more for deep grey matter). Our results reflect the time required for rat brains to equilibrate in a single volume of 50 mL PBS solution at 4 °C. A single volume of PBS was used here to provide a reference for how fast $T_{1}$ and $T_{2}$ stabilize in the simplest case. Evidence from the literature suggests that rehydration at room temperature^14^, ^25^ and/or frequent replacement of the PBS solution^15^ would further reduce the rehydration time. Removal of the brain from the skull prior to rehydration may also decrease rehydration time.

As for the DTI metrics, FA and MD remained generally stable in the white matter of the CC during the 35 day period. A slight decreasing trend was observed in the FA of the cortex, which may be due to an increase in SNR from the longer $T_{2}$ at greater rehydration times, as noisy data may cause an overestimation of FA. A slight decrease was observed in the MD results for the thalamus at 2% PFA. In the literature, a slight trend of increased diffusivity with rehydration has been reported for the immersion‐fixed monkey brain.^2^ This difference may be due to different fixation conditions. It may be possible that, for the immersion‐fixed brain, dehydration occurs in the interval between death and fixation, which is reversed by soaking the sample in PBS. Dehydration may also occur in prolonged periods of immersion in formaldehyde. In the perfusion case, there is no interval between death and fixation, and the time of immersion in fixative is much shorter, so we may be seeing less dehydration, and therefore less change in MD following rehydration.

### 4.2 Gadolinium experiment

The relationship we found between observed relaxation times and Gd‐DTPA concentration in perfusate followed the theoretical model^33^ in line with previous ex vivo MRI studies.^2^, ^40^ Differences between measurements in the three regions of interest in our results were small compared with inter‐sample variation and the effects of changing fixative and gadolinium concentration, suggesting that the same optimization protocol can be applied effectively for white, cortical grey and deep grey matter tissue. Note that the concentration of contrast agent in the tissue at the time of scanning is expected to be less than that used in the perfusate due to both partial penetration of the contrast agent during perfusion, and equilibration between the tissue and PBS solution during rehydration. Rather than an indication of the concentration of gadolinium in the tissue, our results should be interpreted as a practical guide for how much contrast agent to use in the active staining protocol.

Regarding the DTI measurements, FA and MD were generally well preserved in the three regions of interest, for increasing concentrations of Gd‐DTPA. In the CC and thalamus of the 2% model, a slight negative trend in FA was observed. This could be an effect of SNR increasing with contrast agent. The MD results in the grey matter regions for both fixative models also showed a slight decrease. As suggested in Reference,^2^ this may be due to an increase in local magnetic field gradients due to greater magnetic susceptibility effects at higher concentrations of Gd‐DTPA.

The comparison of contrast agents revealed shorter $T_{1}$ and $T_{2}$ in the rats fixed with gadobutrol than Gd‐DTPA, in line with previous studies on blood and plasma.^41^, ^42^, ^43^, ^44^ We observed a higher $T_{2}$‐to‐ $T_{1}$ ratio in the gadobutrol results, suggesting that in future switching from Gd‐DTPA to gadobutrol would further improve SNR efficiency. The difference between the two contrast agent models at the initial point where the concentration in perfusate is 0 mM implies that the 1 mM of contrast agent used in rehydration had a significant effect. The smaller effect of the contrast agent used in perfusate is probably due to partial penetration, as mentioned above.

### 4.3 Fixative concentration

Rats fixed with the lower concentration of fixative, 2% PFA compared with the standard 4%, had longer $T_{1}$ and $T_{2}$, in line with previous studies.^15^, ^23^ Furthermore, rats fixed with 2% PFA required nearly half the time for $T_{2}$ to stabilize in the CC, and less than half the time in the grey matter regions. While the effect of changing the fixative concentration was relatively small compared with that of contrast agent, the shorter rehydration time required and the gains in $T_{2}$, particularly for concentrations of Gd‐DTPA of about 25 mM or less, make it a worthwhile factor to consider.

DTI metrics FA and MD were not affected by the change in fixative concentration in the rehydration experiment, but in the gadolinium experiment FA was lower in the 2% fixative model (mean FA = 0.33, cf. 0.41). This may be due to lower SNR from the higher concentrations of Gd‐DTPA, or from changes in permeability between the two fixation models influencing the amount of gadolinium present in different compartments.

### 4.4 SNR efficiency optimization

Our strategy for SNR efficiency optimization takes into account the effects of fixative concentration, tissue rehydration and contrast agent concentration as well as scanning parameters TE and TR. The first conclusion from the results discussed above is to use a reduced fixative concentration of 2% PFA, and a tissue rehydration sufficiently long for $T_{2}$ to stabilize (in our experiments a minimum of 14, 17 and 20 days for the cortex, CC white matter and thalamus), to prolong $T_{2}$ and maximize SNR efficiency.

Second, we determine the optimal concentration of contrast agent introduced in perfusion to maximize SNR efficiency, in conjunction with TE and TR for diffusion‐weighted spin echo sequences, using our SNR efficiency optimisation tool, available at https://github.com/rachellcb/SNR-Efficiency-Calculator. In practice, TE is the parameter most constrained by factors such as diffusion weighting, acquisition matrix size and gradient strength, while TR is constrained by the total scan time available and the number of diffusion directions required. Contrast agent, on the other hand, can be freely varied. For a given TE, the TR and contrast agent concentration should then be chosen together to maximize SNR efficiency, with an upper limit on TR based on scan time constraints if needed. In our example of tissue optimization for high‐resolution diffusion MRI, we used TE = 26.78 ms, TR = 250 ms and [Gd‐DTPA] = 15 mM. The SNR resulting from a single $T_{2}$‐weighted volume in this optimized tissue sample was double that of a baseline sample prepared with the standard 4% PFA and no gadolinium, all other factors being equal.

The optimization strategies presented in this paper may be used to improve SNR efficiency with any sequence for which the signal is greater with increased $T_{2}$ relative to $T_{1}$. This includes other spin echo sequences such as spin echo EPI as well as the standard pulsed gradient spin echo sequence focused on here. In the case of fast spin echo or gradient echo sequences in which the signal depends on additional variables not considered here, such as flip angle, inversion time and $T_{2}$*, the optimal amount of contrast agent may be different.

The optimal parameters for different studies may also depend on various factors such as b‐value (for diffusion sequences), resolution, gradient strength, field strength and tissue relaxivity. For example, a lower b‐value and lower resolution may be sufficient for certain applications, allowing a shorter TE, and leading to a shorter optimal TR and higher concentration of contrast agent. Scanners with lower gradient strength will generally necessitate a longer TE to achieve the same diffusion weighting, which would correspond to a longer optimal TR and lower concentration of contrast agent, according to our results in Figure 5. At higher field strengths, while more signal is available, $T_{1}$ is generally longer,^45^ so we would expect more gadolinium to be required to achieve maximum SNR efficiency.

In our tissue preparation protocol there were certain factors that were fixed and not included as optimization variables, for example the volume and replacement frequency of the rehydration solution mentioned earlier, or the duration of immersion time in the fixative mixture. $T_{1}$ and $T_{2}$ both decline with immersion time,^46^ so for a shorter immersion time, say two days instead of four, we might expect the optimal concentration of gadolinium to be lower. While the direct application of our SNR efficiency optimization results is limited to cases where protocols and hardware specifications are consistent with ours, these results will nevertheless serve as a useful benchmark for other studies, and a starting point for those undertaking study‐specific tissue optimization.

### 4.5 High‐ and mid‐resolution diffusion imaging

Using optimized tissue preparation, we were able to acquire high‐quality high‐resolution diffusion data. Angular diffusion contrast was high enough to successfully apply HARDI modelling (spherical deconvolution) to resolve crossing fibres and reconstruct white matter tracts anatomically consistent with axonal tracing atlases.^38^, ^39^ Fine anatomical features were visible in the tractograms, diffusion parameter maps and fibre orientation distribution fields. FA values in white matter, which serve as an index of diffusion contrast, were comparable to those reported for other high‐resolution ex vivo diffusion acquisitions in the literature^2^, ^4^, ^47^, ^48^ (0.55 in the middle of the CC and up to 0.7 in other regions). While we did not apply the high‐resolution diffusion protocol to the baseline preparation (no gadolinium, 4% PFA), we predicted that applying this would result in a pre‐denoising SNR in the non‐diffusion volumes of less than 5, i.e., below what is considered acceptable for diffusion modelling. Alternatively, to modify the sequence to achieve equivalent SNR in the baseline case without compromising on spatial resolution or diffusion parameters would require triple the acquisition time.

Other studies have used specialized hardware or advanced pulse sequences to address the challenge of insufficient SNR in high‐resolution ex vivo diffusion imaging. Johnson, Calabrese and colleagues used a custom‐made 30 mm diameter radiofrequency transmit receive coil with 650 mT/m gradients at 7 T to achieve a voxel size of 50 μm, six directions and a *b*‐value of approximately 1500 s/mm ${}^{2}$ in the rat brain.^21^, ^28^, ^30^ Alternative pulse sequences can also reduce scan time without compromising SNR. For example, Aggarwal et al^47^ used a diffusion‐weighted gradient and spin echo sequence with 3000 mT/m gradients at 11.7 T to achieve a voxel size of 55 μm with 12 diffusion directions and a *b*‐value of 1700 s/mm ${}^{2}$ in the mouse brain. In comparison, using our tissue preparation and optimization approach, with a 1000 mT/m gradient set and 39 mm volume coil at 9.4 T, we were able to achieve a voxel size of 78 μm with 30 directions and a *b*‐value of 4000 s/mm ${}^{2}$ in the rat brain.

This shows that with improved tissue preparation it is practical to achieve sufficient SNR, angular diffusion sampling and diffusion contrast for advanced modelling using the standard hardware and pulse sequences available on preclinical MRI systems. Further gains in SNR efficiency could be made by combining the tissue optimization approach described here with improved pulse sequences and hardware, to further push the boundaries of high‐resolution ex vivo diffusion imaging.

Improvements in SNR efficiency can equally be applied to low‐ or mid‐resolution acquisitions in order to improve data quality or decrease scan time. This could be useful for instance in studies where scanning hours are limited, or large numbers of samples are required. The mid‐resolution diffusion‐weighted EPI acquisition was included as an example of a protocol that can be achieved in an overnight scanning session, with a voxel size of 150 μm, 30 directions, *b*= 2000 s/mm ${}^{2}$ and scan time of 9.13 h. This protocol included four signal averages, but the SNR measured was high enough that a single average would provide sufficient SNR for standard DTI measurements in a quarter of the time (2 h, 17 min) if desired.

### 4.6 Histology

The histology data, including stains for cell bodies, microglia and myelin, showed no discernible differences between samples prepared with the standard 4% PFA, the reduced fixative 2% PFA or the optimized preparation with 2% PFA and Gd‐DTPA. These results suggest that it is possible to achieve adequate fixation for histology using 2% PFA. A previous study^49^ shows histology data from mouse brain samples fixed with 4% and 0.5% PFA, the 0.5% PFA sample clearly showing tissue and cell degradation, which were not observed in our 2% PFA sample. Compatibility of tissue prepared with contrast agent for histology has also been shown previously.^50^, ^51^, ^52^

### 4.7 Conclusion

In this study, we described the effects of tissue preparation factors on relaxivity and diffusivity of the CC, cortex and thalamus in fixed rat brain tissue, and how these can be combined to optimize SNR efficiency. The approach we propose is to use a fixative concentration of 2% PFA, to rehydrate the tissue for at least 20 days and to optimize the concentration of contrast agent depending on the minimum possible TE and corresponding optimal TR within one's time constraints, using our results as a guide. The approach used here increased SNR by a factor of more than 2.5 compared with a standard preparation, and diffusion properties FA and MD were sufficiently preserved. We illustrated the application of this tissue optimization approach with one high‐ and one medium‐resolution diffusion dataset, with examples of tractography with high anatomical detail and multi‐fibre modelling. In conclusion, our strategy will allow researchers to achieve faster acquisitions of high‐quality, high‐resolution data for advanced diffusion analyses using a standard preclinical setup.

## AUTHOR CONTRIBUTIONS

**Rachel L. C. Barrett:** conceptualization, methodology, software, formal analysis, investigation, writing—original draft, visualization, project administration. **Diana Cash:** resources, writing—review & editing. **Camilla Simmons:** resources. **Eugene Kim**: resources, writing—review & editing. **Tobias Wood:** software, writing—review & editing. **Richard Stones:** writing—review & editing. **Anthony Vernon:** writing—review & editing. **Marco Catani:** funding acquisition. **Flavio Dell'Acqua:** conceptualization, methodology, software, investigation, writing—review & editing, supervision.

## Supporting information

nbm4866‐sup‐0001‐RBarrett.pdf

## Data Availability

The data that support the findings of this study are available at https://doi.org/10.6084/m9.figshare.21629894 and from the corresponding author upon reasonable request., and the code for SNR efficiency optimisation is available at https://github.com/rachellcb/SNR-Efficiency-Calculator
