# Supplementary material for: Tissue optimization strategies for high‐quality ex vivo diffusion imaging
Source: NMR Biomed. 2022 Dec 4;36(3):e4866. doi: 10.1002/nbm.4866 (PMC10078604; doi:10.1002/nbm.4866)
Supplement: Supplementary file 1 — nbm4866‐sup‐0001‐RBarrett.pdf [file NBM-36-0-s001.pdf]

Supplementary Information

Tissue Optimisation Strategies for High Quality Ex Vivo Diffusion Imaging

Rachel L. C. Barrett, Diana Cash, Camilla Simmons, Eugene Kim, Tobias C. Wood, Richard Stones, Anthony C. Vernon, Marco Catani, Flavio Dell'Acqua

This PDF file includes:

Figs. S1 and S2

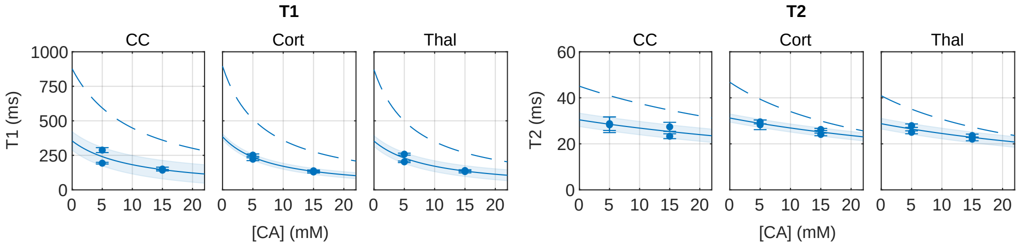

Figure S1: Comparison of relaxation time vs concentration curves for Gd-DTPA (dashed line) and gadobutrol (data points and solid line). Graphs show how T1 and T2 vary with concentration of contrast agent (CA) used in perfusion. A constant 1 mM CA is used in the rehydration stage for both models. The differing initial values between the models represent the effect of rehydrating the tissue in 1 mM CA, in absence of CA in the perfusate. Data points and error bars represent mean values in the corpus callosum (CC), thalmus (Thal) and cortical grey matter (Cort), for the gadobutrol model. Data points and error bars for the Gd-DTPA model are omitted here for simplicity but shown in Figure 3. Lines of best fit are included.

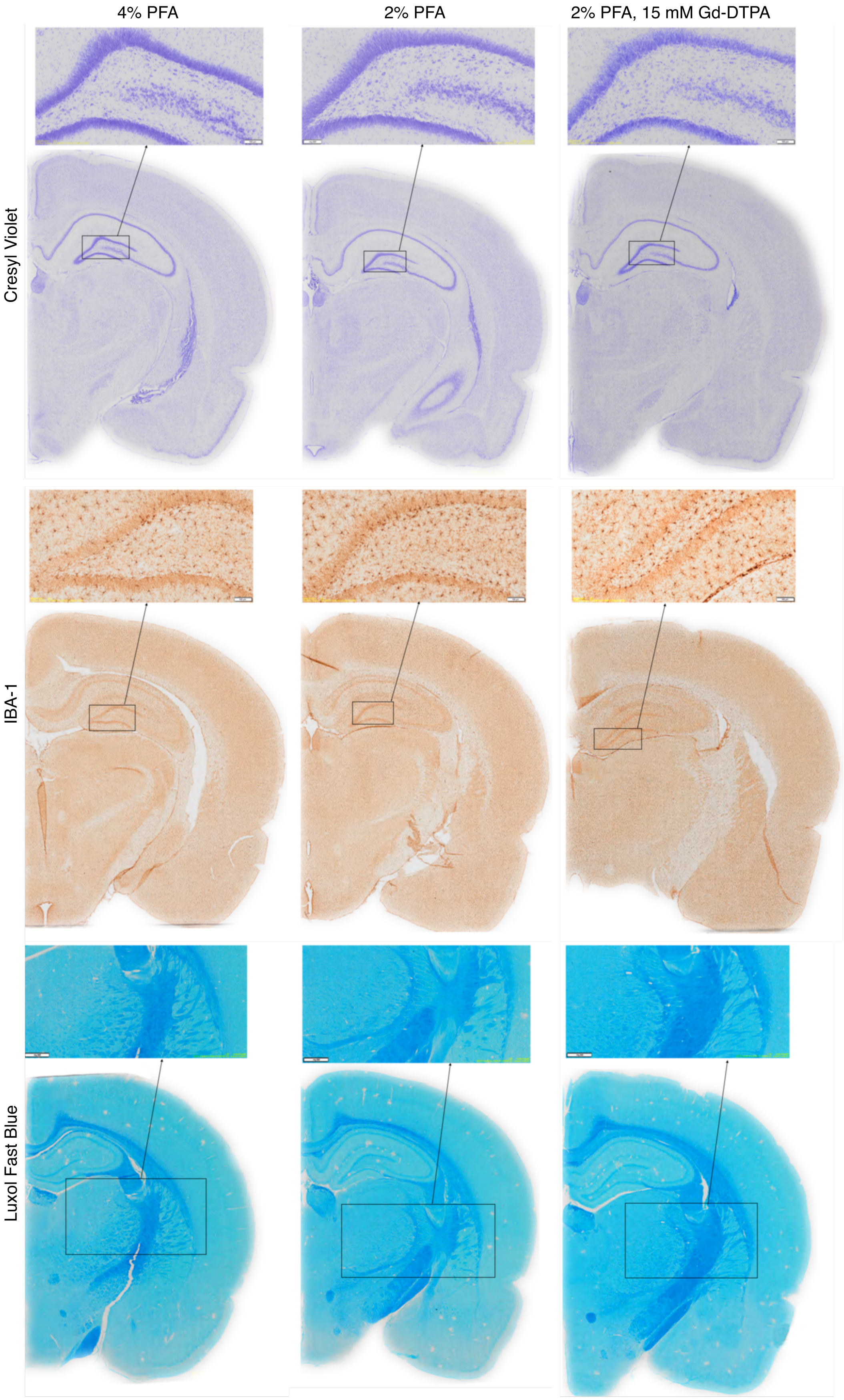
4% PFA
2% PFA
2% PFA, 15 mM Gd-DTPA
Cresyl Violet
IBA-1
Luxol Fast Blue

Figure S2. Histology showing no differences between different tissue preparations. The different preparations are compared in columns from left to right: 4% PFA; no contrast agent (standard protocol), 2% PFA, no contrast agent, 2% PFA, 15 mM Gd-DTPA added during perfusion (optimised protocol). Each sample was rehydrated in PBS, or PBS with 1 mM Gd-DTPA in the optimal case. From top to bottom the stains used are Cresyl Violet, IBA-1 antibody and Luxol Fast Blue. The inserts highlight neurons and microglia in the hippocampus, and white matter in the internal capsule and striatum.
